# Procyanidin B2 inhibits angiogenesis and cell growth in oral squamous cell carcinoma cells through the vascular endothelial growth factor (VEGF)/VEGF receptor 2 (VEGFR2) pathway

**DOI:** 10.1080/21655979.2022.2033013

**Published:** 2022-02-27

**Authors:** Qiurong Sun, Taiyang Zhang, Qingchun Xiao, Bingxin Mei, Xingwang Zhang

**Affiliations:** a Department of Stomatology, The First Affiliated Hosital of Gannan Medical University, Ganzhou, Jiangxi, China; b Department of Oral Surgery, Daqing Longnan Hospital, Daqing, Heilongjiang, China

**Keywords:** Procyanidin B2, angiogenesis, cell growth, oral squamous cell carcinoma, VEGF/VEGFR2 pathway

## Abstract

This study aimed to explore the therapy role of procyanidin B2 (PB2) in inhibiting angiogenesis and cell growth in oral squamous cell carcinoma. After oral mucosa epithelial cell (OMEC) and human oral squamous cell carcinoma (OSCC) cell line (SCC-25) were treated with PB2 or SCC-25 were treated with PB2 and rhVEGF, cell counting kit-8 (CCK-8) assay was used to determine the cell viability. The apoptosis, migration, invasion and angiogenesis of SCC-25 after indicated treatment were detected by Tunel, wound healing, transwell and tube formation assays. The protein expression related to apoptosis, metastasis and epithelial-mesenchymal transition (EMT) and changed expression of vascular endothelial growth factor (VEGF)/VEGF receptor 2 (VEGFR2) signaling was analyzed by Western blot. As a result, PB2 inhibited viability, invasion, migration and EMT and promoted apoptosis of SCC-25 cells. In addition, PB2 inhibited VEGF/VEGFR2 signaling and tumor itangiogenesis in OSCC. As expected, activation of VEGF/VEGFR2 signaling suppressed the effect of PB2 on growth and metastasis of OSCC cells. In conclusion, PB2 inhibited the VEGF/VEGFR2 pathway to suppress the angiogenesis and cell growth of SCC-25 cells.

## Graphical abstract

**Figure:**
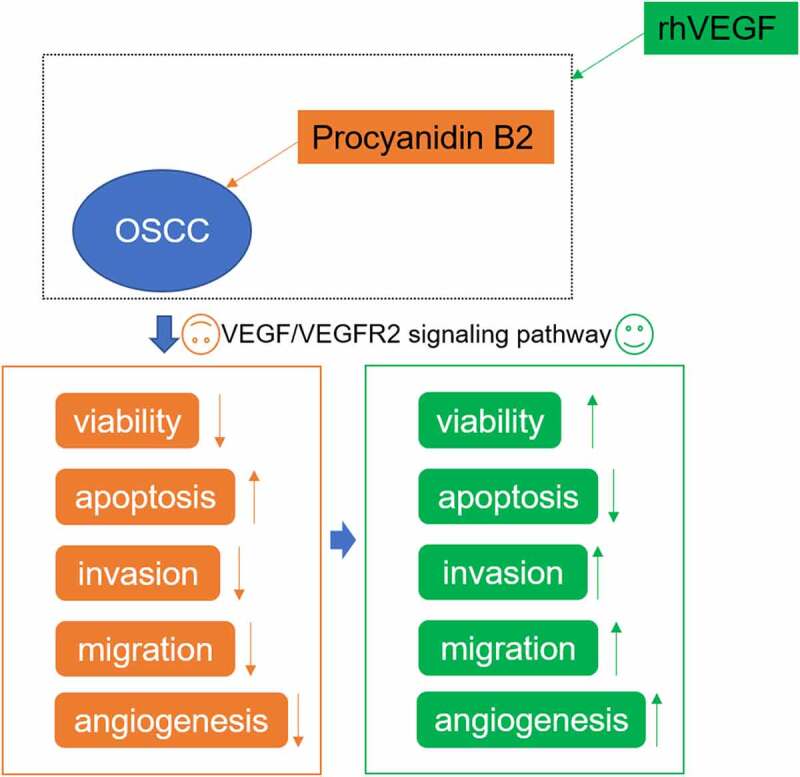

## Introduction

The incidence of head and neck malignant tumors is increasing worldwide, and has risen to the sixth place in all tumors. Head and neck malignant tumors often occur in patients’ throat, pharynx, nasal cavity, oral and maxillofacial regions, etc [1]. The latest study by the International Agency for Research on Cancer (IARC) shows that in 2012, 529,500 head and neck malignant tumors occur worldwide, accounting for 3.8% of all cancer cases. Due to the changing global population structure, the incidence of head and neck malignant tumors is expected to increase to 6.2% by 2035, approximately 856,000 cases [2]. Among different types of head and neck malignancies, oral squamous cell carcinoma (OSCC) is the most harmful and has the highest incidence, accounting for more than 90% of all oral cancer patients [3]. Although the strategy and level of multidisciplinary comprehensive sequence therapy in OSCC have been greatly developed, the 5-year survival rate of patients has not been significantly improved and remains at about 60% [4]. In view of this, it is necessary to find new therapies for the clinical treatment of OSCC and improve the clinical prognosis of patients.

Procyanidin is a common natural crosslinking agent with strong antioxidant capacity. Meanwhile, studies have shown that procyanidin compounds have anti-cancer activity, which can inhibit the proliferation of cervical cancer cells [5] and induce cell apoptosis by activating c-Jun N-terminal kinase (JNK) in esophageal adenocarcinoma [6]. Studies have shown that GSP (grape seed proanthocyanidins, a mixture) can partially inhibit the malignant development of OSCC cells through the p53 pathway [7], but the effect of its single component on OSCC remains unknown.

Procyanidin B2 (PB2) is an important single component of procyanidin. PB2 is often used to calibrate the content of procyanidin in grape seed extract. Previous studies have shown that it can promote autophagy and apoptosis of colorectal cancer cells through phosphatidylinositol-4,5-bisphosphate 3-kinase (PI3K)/protein kinase B (AKT) signaling pathway [8], and has cytotoxicity to breast cancer cells [9]. Therefore, it is speculated that it can play an important anticancer effect in OSCC. STITCH database analysis showed that PB2 could participate in VEGF signaling pathway. Through literature review, it was found that PB2 inhibited VEGFR2 phosphorylation and inhibited endothelial cell growth and motility [10]. VEGF/VEGFR2 is a key pathway for angiogenesis in tumors, and some studies have shown that inhibition of angiogenesis in OSCC can limit tumor progression [11].

It was hypothesized that PB2 could inhibit angiogenesis and cell growth in OSCC through the VEGF/VEGFR2 pathway. In the present study, different concentrations of PB2 were used to treat OSCC cells and the viability, apoptosis, invasion and migration of OSCC cells were analyzed. Then, the changes of VEGF/VEGFR2 pathway affected by PB2 were also determined. Finally, rhVEGF was used to verify the role of VEGF/VEGFR2 pathway in PB2 regulating OSCC cells.

## Materials and methods

### Cell culture and treatment

Oral mucosa epithelial cell (OMEC) was obtained from Procell (Wuhan, China) and human OSCC cell line (SCC-25) was purchased from American Type Culture Collection (ATCC). OMEC was cultured in the completed culture medium for human oral epithelial cells (Procell). SCC-25 cells were cultured in dulbecco’s modified eagle medium (DMEM)/F12 containing 10% FBS, 1% P/S and 400 ng/mL hydrocortisone. All cells were incubated at 37°C with 5% CO_2_. OMEC and SCC-25 cells (passage 3) were treated with procyanidin B2 (PB2) at different concentrations (0 μM, 1 μM, 5 μM and 10 μM) for 48 h. Another experiment is that SCC-25 cells were pre-treated with 10 μM PB2 for 48 h and then treated with 100 ng/ml recombinant human VEGF (rhVEGF, PeproTech, 100–20-2) for 2 h.

### Bioinformatics

The STITCH DataBase (version 5.0; http://stitch.embl.de/) is a database that can predict the interactions between chemicals and proteins [12].

### Cell counting kit-8 (CCK-8) assay

OMEC and SCC-25 cells in each group were laid on 96-well plates at the density of 1 × 10^3^ cells per well. After gently mixed and indicated treatment, cells were cultured in the incubator at 37°C. The 96-well plate was taken out at 48 h and each well was added with 10 μL CCK8 solution for 1 h. Finally, the 96-well plate was placed on a microplate reader to detect the optical density (OD) value at 450 nm.

### Tunel assay

At the end of each experiment, SCC-25 cells were washed three times with pre-cooled PBS to remove suspended cytoplasm and culture medium. Then, cells were placed in 4% paraformaldehyde for fixation for 15 min at 4°C and stained with 4-6-diamidino-2-phenylindole (DAPI) for 10 min in dark. After PBS washing for three times, the apoptosis of SCC-25 cells was observed under an optical microscope (Olympus Corporation, Tokyo, Japan).

### Western blot analysis

SCC-25 cells in each group were lysed with an appropriate amount of RIPA lysate for 30 min, centrifuged 12,000 r/min at 4°C for 10 min, and the supernatant was collected. Total protein in the cells was extracted with total protein extraction kit, and protein content was determined with BCA protein quantitative kit. Protein lysates were separated by 10% sodium dodecyl sulfate polyacrylamide gel electrophoresis (SDS-PAGE) and transferred to polyvinylidene fluoride (PVDF) membranes. The membranes were then blocked with 5% milk at room temperature for 2 h, followed by incubated with primary antibodies Bax, Bcl-2, MMP2, MMP9, Vimentin, E-cadherin, VEGF, p-VEGFR2, VEGFR2 and GAPDH at 4°C overnight. The next day, phosphate buffered solution (PBST) washed the membranes, and then incubated with horseradish Peroxidase (HRP)-labeled goat anti-rabbit IgG secondary antibody at room temperature for 2 h. The polymerase chain reaction-enhanced chemiluminecence (ECL) was applied to observe the protein bands and gray values were analyzed by using ImageJ software (v1.8; National Institutes of Health).

### Wound healing assay

SCC-25 cells in each group were evenly laid on 6-well plates at the density of 5 × 10^5^ cells per well. The cell monolayer was scratched with 200 μl pipette tip and assisted by a ruler. PBS was added to well to wash away the cell fragments, and then 1% serum contained medium was added. The cells were placed under an optical microscope (Olympus Corporation) and photographed, which was recorded as 0 h at this time. The cells were cultured at 37°C and 5% CO_2_ incubator for 24 h, and the cell movement was observed under an inverted microscope and photographed.

### Transwell assay

SCC-25 cells in each group were incubated into the upper chamber of matrigel coated polyethylene terephthalate membrane (40 μL/well, 8 μm, Corning, USA) at the density of 5 × 10^5^ cells/well. The lower chamber was added with 600 μL medium containing 10% FBS. It was placed in the incubator for normal culture at 37°C for 24 h. The medium was discarded and cells inside the upper chamber were wiped with a swab. The invasive cells at the bottom of the upper chamber were fixed in methanol for 1 min and then stained with 500 μL 0.1% crystal violet solution diluted by PBS at room temperature at 15 min in dark. The invasion was observed under an optical microscope (Olympus) and photographed for counting.

### Tube formation assay

SCC-25 cells in each group were inoculated into the 48-well plate coated with Matrigel (BD Biosciences; 200 μl/well) and added with the culture medium. The tubular arrangement and integrity of cells were observed during cell culture. Cell culture was terminated after 24 h. The tube formation of the SCC-25 cells was assessed with a phase-contrast microscope (Carl-Zeiss).

### Statistical analysis

GraphPad Prism 8 (GraphPad Inc, USA) was used for data analysis. Quantitative data were presented as mean ± SD of at least three independent experiments. Differences between two groups were analyzed by the Student’s t test and differences among multiple groups were analyzed by the one-way ANOVA with Tukey’s post hoc test. P < 0.05 was considered to be statistically significant.

## Results

### PB2 inhibited viability and promoted apoptosis of OSCC cells

**Figure 1.**
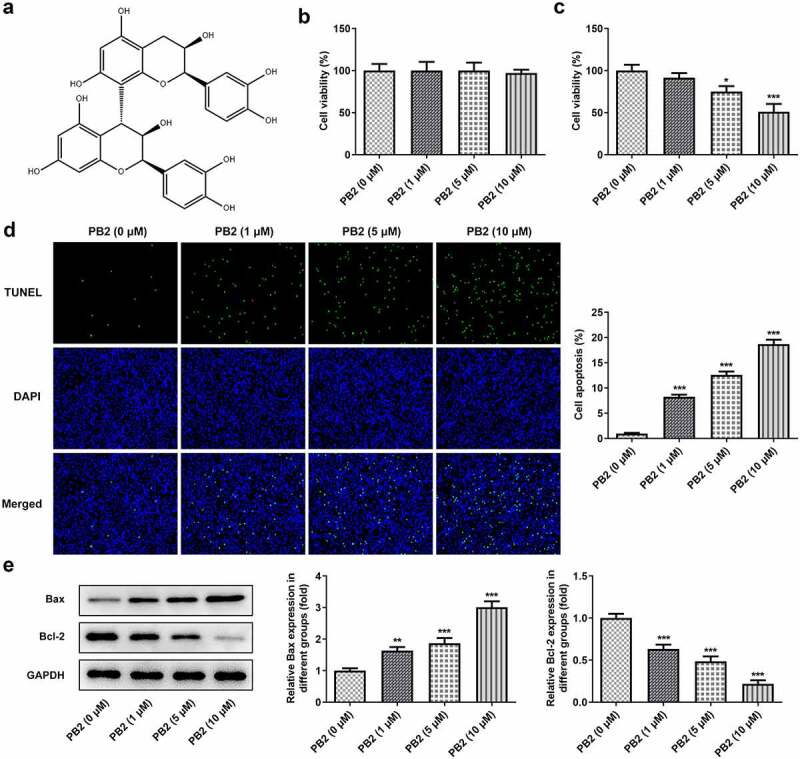
PB2 inhibited viability and promoted apoptosis of OSCC cells. (a) The chemical structure of PB2. (b) The viability of oral mucosa epithelial cell (OMEC) treated with PB2 was detected by CCK-8 assay. (c) The viability of human OSCC cell line (SCC-25) treated with PB2 was detected by CCK-8 assay. (d) The apoptosis of SCC-25 cells treated with PB2 was analyzed by Tunel assay. (e) The expression of apoptosis related proteins in SCC-25 cells treated with PB2 was determined by Western blot. *P < 0.05, **P < 0.01 and ***P < 0.001 vs. PB2 (0 μM) group.

The chemical structure of PB2 is shown as Figure 1(a). The effect of different concentrations of PB2 on the viability of OMEC and OSCC cell was confirmed by CCK-8 assay. The Tunel assay and Western blot were applied to confirm the effect of different concentrations of PB2 on the OSCC cell apoptosis. With increase of PB2 concentration, the viability of OMEC was not obviously changed (Figure 1(b)). The inhibition effect of OSCC cell viability was gradually enhanced with the increasing concentration of PB2 (Figure 1(c)), and so the apoptosis of OSCC cells was promoted (Figure 1(d)). The Bax expression was gradually increased while Bcl-2 expression was gradually decreased by PB2 ranging from 0 to 10 μM (Figure 1(e)).

### PB2 inhibited invasion, migration and epithelial-mesenchymal transition (EMT) of OSCC cells

**Figure 2.**
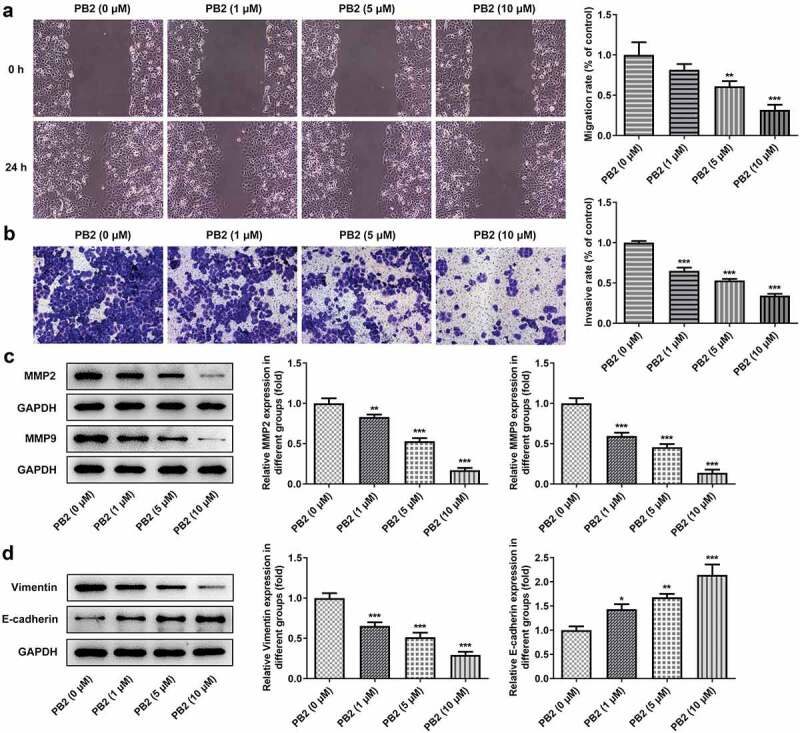
PB2 inhibited invasion, migration and epithelial-mesenchymal transition (EMT) of OSCC cells. The migration (a) and invasion (b) of SCC-25 cells treated with PB2 were detected by wound healing assay and transwell assay. The expression of metastasis associated proteins (c) and EMT related proteins (d) was analyzed by Western blot. *P < 0.05, **P < 0.01 and ***P < 0.001 vs. PB2 (0 μM) group.

The invasion and migration of OSCC cells treated with different concentrations of PB2 were analyzed by transwell and wound healing assays, accompanied with the Western blot. The invasion and migration of OSCC cells was restrained gradually when the concentration of PB2 was changed from 0 to 10 μM (Figure 2(a-b)), and so the expression of MMP2 and MMP9 was also decreased (Figure 2(c)). PB2 suppressed the expression of Vimentin and promoted the expression of E-cadherin with the PB2 concentration increased (Figure 2(d)).

### PB2 inhibited VEGF/VEGFR2 signaling and tumor angiogenesis in OSCC

**Figure 3.**
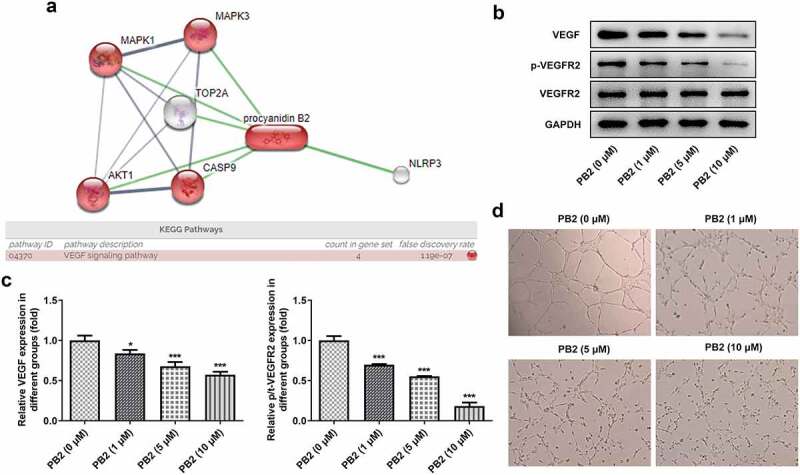
PB2 inhibited VEGF/VEGFR2 signaling and tumor angiogenesis in OSCC. (a) The related genes of PB2 were analyzed by STTICH database. (b/c) The expression of VEGF/VEGFR2 signaling in SCC-25 cells treated with PB2 was analyzed by Western blot. (d) The angiogenesis of SCC-25 cells treated with PB2 was detected by tube formation assay. *P < 0.05 and ***P < 0.001 vs. PB2 (0 μM) group.

As shown in Figure 3(a), STITCH database indicated that there were six proteins interacting with PB2. According to KEGG pathways analysis in STITCH database, four proteins were found to be enriched in VEGF signaling pathway. After PB2 treatment, the expression of VEGF signaling pathway related proteins was detected by Western blot and the angiogenesis was observed by tube formation assay in OSCC cells. The expression of VEGF and p/t-VEGFR2 was suppressed gradually by the increase of PB2 (Figures 3(b-c)). In addition, PB2 also inhibited the endothelial tube formation (Figure 3(d)).

### Activation of VEGF/VEGFR2 signaling reduced the effect of PB2 on growth and metastasis of OSCC cells

**Figure 4.**
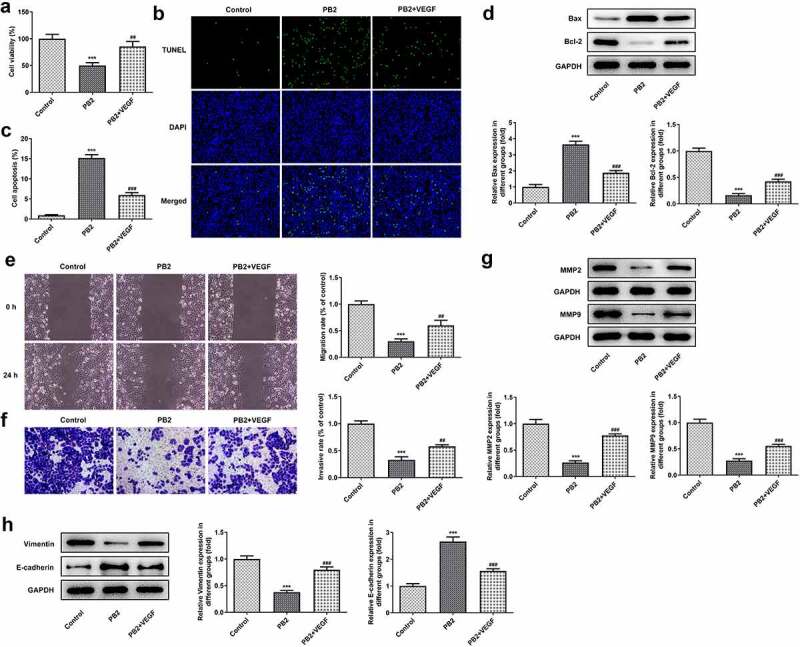
Activation of VEGF/VEGFR2 signaling reduced the effect of PB2 on growth and metastasis of OSCC cells. (a) The viability of SCC-25 cells treated with PB2 with or without VEGF was detected by CCK-8 assay. The apoptosis (B/C) and related proteins (d) in SCC-25 cells treated with PB2 with or without VEGF were respectively analyzed by Tunel assay and Western blot. The migration (e) and invasion (f) of SCC-25 cells treated with PB2 with or without VEGF were detected by wound healing assay and transwell assay. The expression of metastasis associated proteins (g) and EMT related proteins (h) was analyzed by Western.

The effect of rhVEGF on the PB2 regulating the viability, apoptosis, invasion and migration was analyzed by CCK-8, Tunel, transwell and wound healing assays, and corresponding protein expression was detected by Western blot. rhVEGF could improve the viability (Figure 4(a)) and inhibit the apoptosis of PB2-treated OSCC cells (Figures 4(b-c)). After addition of rhVEGF treatment, the expression of Bax was inhibited while the expression of Bcl-2 was increased in PB2-treated OSCC cells (Figure 4(d)). rhVEGF made the migration and invasion increased (Figures 4(e-f)) and correspondingly promoted the expression of MMP2 and MMP9 in PB2-treated OSCC cells (Figure 4(g)). In addition, rhVEGF upregulated the Vimentin expression and downregulated the expression of E-cadherin in in PB2-treated OSCC cells (Figure 4(h)).

## Discussion

PB2 is the most common type of procyanidins, which can reduce the risk of diabetes, lipid metabolism disorders, cancer and other diseases [13,14]. PB2, in particular, has shown anticancer activity in various types of cancer, including breast cancer [9], prostate cancer [15], gastric cancer [16] and colorectal cancer [8]. Our finding indicated that PB2 could inhibit the viability and promote the apoptosis of OSCC cells, which was consistent with the previous findings showing the anticancer activity of PB2.

The matrix metalloproteinases (MMPs) family is considered to play an important role in tumor invasion and migration by disrupting histological barriers that inhibit tumor cell invasion to other regions [17]. Previous study has shown that MMP-2 and MMP-9 play important roles in tumor invasion and migration [18]. Overexpression of MMP-2 and MMP-9 can enhance the invasion and migration of tumor cells. In this study, the protein expression level of MMP-2 and MMP-9 in SCC-25 cells was significantly decreased with the intervention of PB2, indicating that the migration and invasion ability of OSCC cells were inhibited.

EMT refers to the transformation of epithelial cells into mesenchymal cells without polarity and intercellular adhesion, which is closely related to the invasion and metastasis of malignant tumors [19]. During EMT, the expression of E-cadherin was decreased, while the expression of N-cadherin and vimentin was increased [20]. Studies have shown that EMT is closely related to the invasion and metastasis of various tumors [21–23]. Here, we found that the expression of E-cadherin was increased, while the expression of vimentin was decreased after PB2 treatment. And, the migration and invasion ability of OSCC cells were also suppressed by PB2 treatment.

Malignant tumors need nutrients and oxygen to survive and proliferate, so they need to grow near blood vessels in order to enter the bloodstream. The more new blood vessels in a tumor, the more malignant the tumor is. Tumor cell growth, invasion and metastasis are achieved through angiogenesis. In addition, angiogenic factors are existed in tumor microenvironment and endodermal cells are induced to proliferate and form new tumor blood vessels [24,25]. The VEGF family is a group of key proteins associated with angiogenesis pathways. Both biological and clinical evidence suggest that blocking VEGFR2 is a promising approach to inhibit tumor-induced angiogenesis [26]. VEGF is an angiogenic promoter secreted by tumor cells and can mediate angiogenesis through its receptor VEGFR2 [26,27]. Previous study indicated that PB2 could inhibit the VEGFR2 phosphorylation and inhibited endothelial cell growth and motility [10]. Therefore, the present study showed that PB2 suppressed the angiogenesis in OSCC cells by inactivating the VEGF/VEGFR2 signaling pathway. And, rhVEGF could reverse the effect of PB2 to promote the viability, invasion and migration of OSCC cells.

## Conclusion

It was found that PB2 inhibited the VEGF/VEGFR2 pathway and therefore suppressed the cell growth and angiogenesis, which was reversed by rhVEGF. Though all the current in vitro studies here suggested that PB2 had an anti-angiogenic property, more in vivo studies were still necessary to confirm its inhibiting effect on vascular tube formation in OSCC. In addition, whether PB2 is directly involved in VEGF synthesis needs to be confirmed in our future study.
